# A Cognitive Interview Study to Evaluate the Feasibility of a Chinese Version of Digital Cognitive Testing

**DOI:** 10.1002/alz70857_101049

**Published:** 2025-12-25

**Authors:** Bin Huang, Duong Huynh, Mary Patterson

**Affiliations:** ^1^ BrainCheck Inc., Austin, TX, USA

## Abstract

**Background:**

Chinese‐Americans are one of the fastest‐growing minority populations in the US, increasing 53% (4.0 to 6.1 million) from 2010 to 2020. In 2022, 14.3% of this population was aged 65+ years, placing them at increased risk of developing dementia. Over 74.5% of older Chinese‐Americans are first‐generation immigrants, most of whom lack the English proficiency needed to take commonly available cognitive tests. This highlights a critical need for cognitive assessments that are linguistically and culturally adapted for older Chinese‐Americans.

**Method:**

Two bilingual translators, fluent in both Chinese and English, independently translated BrainCheck Assess (BC‐Assess)—a battery of evidence‐based digital cognitive assessments—into Simplified Chinese (C‐BC‐Assess). A cognitive interview study was conducted with five healthy older Chinese adults to evaluate the usability of C‐BC‐Assess. Each participant completed the test in person on an iPad with the assistance of a research assistant. The “think‐aloud” method was used to encourage participants to verbalize their thoughts, questions, and any challenges they encountered in real time while completing the test battery. Participants were reassured that the study focused on usability, allowing them to take the test at their own pace without concern for their final scores. After completing the test, participants were interviewed about their overall experiences.

**Result:**

The five participants, including three females, ranged in age from 60 to 73 years. All had attained either a college degree (*n* = 3) or a graduate degree (*n* = 2). While all participants spoke Mandarin, three were more familiar with Traditional Chinese. Their self‐reported English proficiency levels varied: one basic, two intermediate, and two proficient. All participants rated the usability of C‐BC‐Assess highly, with an average score of 1.6 out of 5 (where lower scores indicate better usability). Participants familiar with Traditional Chinese reported no difficulties using the Simplified Chinese version of BC‐Assess. Additionally, participants identified a few areas in the instructions and assessments that require further cultural adaptations.

**Conclusion:**

This pilot study demonstrated the feasibility of developing a Chinese version of BC‐Assess based on the existing English version. It also highlighted areas requiring cultural adaptations beyond direct translations to ensure usability and relevance.

